# Multi-Scale Spatiotemporal Attention Network for Early Warning of Lithium-Ion Battery Thermal Runaway

**DOI:** 10.3390/s26103083

**Published:** 2026-05-13

**Authors:** Yangyang Liu, Guoli Li, Qunjing Wang

**Affiliations:** School of Electrical Engineering and Automation, Anhui University, Hefei 230601, China; z23111016@stu.ahu.edu.cn (Y.L.); wangqunjing@ahu.edu.cn (Q.W.)

**Keywords:** battery thermal runaway, early warning, multi-scale spatiotemporal attention network, feature engineering, adaptive fusion

## Abstract

Lithium-ion battery thermal runaway has become a key safety hazard restricting the development of electric vehicles. Early precursor signals of thermal runaway are characterized by multi-scale features, weak signal strength and spatial coupling, posing significant challenges for traditional methods in achieving accurate early warning. To solve this problem, a multi-scale spatiotemporal attention network (MSTA-Net) is proposed for battery thermal runaway early warning. First, a systematic feature engineering process is designed, including signal denoising, normalization processing and multi-level feature construction, to fully extract discriminative information from voltage and temperature signals. Then, the MSTA-Net architecture is constructed, which includes three parallel feature extraction branches: local fine perception branch based on 1D depthwise separable convolution to capture transient anomalies, a temporal evolution modeling branch based on bidirectional gated recurrent units to learn long-term trends, and a global spatial dependence branch based on a graph attention network to model the spatial propagation of thermal runaway. Finally, an adaptive fusion gate is designed to dynamically fuse the features of each branch according to the input context. The experimental results on the self-built battery thermal runaway dataset show that the proposed MSTA-Net achieves a recall rate of 98.7%, an average early warning time of 115 s and a false alarm rate of 0 times/h. Compared with traditional machine learning and deep learning models such as Random Forest, LSTM and Transformer, the model has significant advantages in early warning accuracy, timeliness and robustness. Ablation experiments verify the effectiveness of each component of the MSTA-Net. The proposed method can provide reliable early warning of thermal runaway only by using the existing voltage and temperature sensors of the battery management system, which has important engineering application value.

## 1. Introduction

Driven by the global dual carbon strategic goals of “carbon peak” and “carbon neutrality”, the automotive industry is focusing on electrification and intelligence, taking them as the core to accelerate the pace of transformation. New energy electric vehicles (EVs) take the lead, as they are important carriers of the dual carbon goals and their market popularity continues to rise. In 2025, global sales of electric vehicles exceeded 20 million units, reaching as high as 20.154 million units, accounting for 23.5% of global new car sales. From this, it can be seen that new energy vehicle market is growing rapidly [1]. As the “heart” of electric vehicles, power batteries directly affect the performance, safety and service life of vehicles. However, with the continuous improvement of battery energy density and the popularization of fast charging technology, the thermal safety problem of batteries has become increasingly prominent [2]. Thermal runaway (TR) is a catastrophic phenomenon of lithium-ion batteries, which refers to the exponential temperature rise caused by the imbalance between heat generation and heat dissipation inside the battery under abuse conditions, accompanied by flammable gas emission, fire and even explosion [3]. Statistics show that more than 60% of public electric vehicle fire accidents are directly related to battery thermal runaway [4,5], which not only causes serious casualties and property losses, but also affects public trust in electric vehicle safety.

The new national standard GB38031-2025 which will take effect in 2026 has stricter requirements for thermal diffusion: after a single battery in the battery pack or system triggers thermal runaway due to internal short circuit, it shall not catch fire or explode, and a thermal event alarm signal shall be provided no later than 5 min after the occurrence of thermal runaway [6]. This puts forward higher requirements for the timeliness and accuracy of battery thermal safety early warning and control. At present, the thermal safety management mode of mainstream battery management systems (BMSs) mostly adopts rule-based fixed threshold judgment, which has inherent limitations [7]: (1) Early warning lag: the fixed threshold is usually set based on engineering experience, which can only respond to obvious abnormalities in the middle and late stages of thermal runaway, and it is difficult to capture early weak precursor signals; (2) Shallow feature utilization: it only uses single-scale features such as absolute temperature value and simple temperature rise rate, ignoring the multi-scale temporal characteristics and spatial coupling relationship of signals; (3) High cost of additional sensors: some studies try to improve early warning reliability by adding smoke, pressure and gas sensors, but this increases system cost and complexity, and faces challenges in long-term stability under vehicle conditions [8,9,10,11,12]. Recently, deep-learning-based methods have been explored for thermal runaway early warning, including multi-scale spatiotemporal attention networks [13], graph convolutional networks with self-attention for battery pack fault diagnosis [14] and physics-informed transformers for early detection under varying operating conditions [15]. 

Specifically, we clarify that while deep learning models like LSTM, GRU and CNN-LSTM have advanced sequence modeling, they often treat cells independently and lack an inherent mechanism to simultaneously capture the multi-scale temporal dynamics (from sub-second transients to minute-long evolutions) and the structural spatial dependencies (thermal/electrical propagation paths) inherent in a battery pack. This results in suboptimal feature representation and limits early warning capability under complex conditions. Furthermore, more complex architectures like Transformers, while powerful, are computationally intensive and can be prone to overfitting on small, noisy battery datasets. Our revised text now clearly identifies the gap: the critical need for a neural architecture that can efficiently and explicitly model multi-scale temporal and spatial features in a unified and synergistic framework. To address the above problems, this study proposes a multi-scale spatiotemporal attention network (MSTA-Net) for battery thermal runaway early warning. The core innovation lies in the design of a “parallel specialized processing + adaptive dynamic fusion” architecture, which realizes the comprehensive modeling of transient anomalies, long-term trends and spatial propagation features of thermal runaway precursor signals. The main contributions of this paper are as follows:

(1) Feature Engineering: Unlike previous methods which typically use raw data or simple statistical features, we have designed a systematic feature engineering framework that explicitly constructs multi-scale features (transient, statistical, spatial correlation), providing the network with input of higher information density.
(2) Network Architecture: Unlike previous methods that focus on a single scale (such as using only temporal models like LSTM/GRU), or using simple serial fusion (like CNN-LSTM), our proposed MSTA-Net adopts a novel parallel-adaptive architecture. It consists of three specialized branches, controlled by an adaptive fusion gate, achieving unprecedented collaborative modeling of multi-scale spatiotemporal features.
(3) Validation: Unlike previous studies that usually validate in limited conditions, we have rigorously tested MSTA-Net under various unseen abuse conditions (heating, overcharging, pinching) to prove its robustness and generalization ability.

## 2. Methods

### 2.1. Dataset Construction

#### 2.1.1. Experimental Setup

The experimental data used in this study were collected from the thermal runaway test of a 138.5 Ah lithium iron phosphate (LFP) battery pack. The battery pack adopts a 1P114S configuration, including a battery management system (BMS), a thermal management system (BTMS) and an electrical system. The BMS uses NXP S32K344 as the main control chip and NXP MC33774 as the AFE chip, which can collect voltage and temperature signals at a sampling frequency of 1 Hz. The thermal management system adopts a liquid cooling scheme, including water nozzles, pipelines and liquid cooling plates.

**Figure 1:**
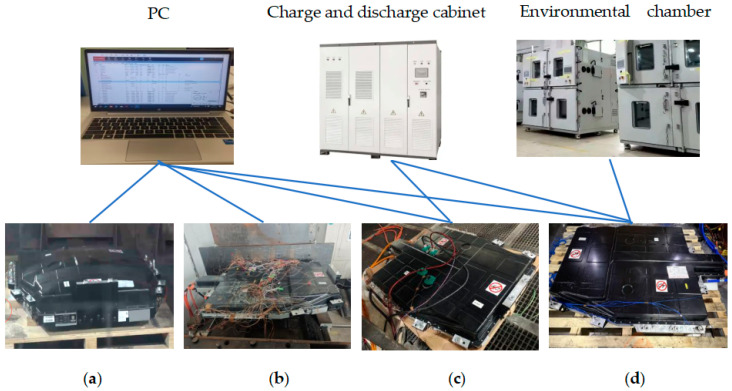
Schematic diagram of the comprehensive test platform for thermal characteristics of power battery packs: (**a**) TR by heating; (**b**) TR by nail; (**c**) TR by overcharge; (**d**) TMP test. TR—Thermal Runaway; TMP—Thermal Management Performance.

Thermal runaway tests were carried out under three trigger conditions: heating, overcharging and nail. The specific test schemes are as follows: (1) Heating trigger: use a 600 W heating plate to heat the target battery until thermal runaway occurs, and record the temperature and voltage signals of the target battery, adjacent batteries and proximate batteries; (2) Overcharging trigger: continuously charge the target battery at 1 C (137 A) until thermal runaway occurs or the charging voltage reaches 150% of the cut-off voltage; (3) Nail trigger: use a steel needle with a diameter of 8 mm and a cone angle of 30° to pierce the target battery at a speed of 1 mm/s, with a piercing depth of 90% of the battery thickness. The environmental chamber is used to conduct charge and discharge tests at different rates and temperatures, as well as cycle tests, to obtain thermal performance data under normal working conditions. The test platform is shown in Figure 1.

#### 2.1.2. Dataset Division

The dataset includes normal working condition data and thermal runaway test data. The normal working condition data includes charging and discharging cycles, static storage and other scenarios under different rates (0.33 C–3 C) and different temperatures (−20–50 °C). The thermal runaway test data includes 4 groups of heating trigger tests, 1 group of overcharging trigger tests and 1 group of nail trigger tests, covering the complete process from normal state to thermal runaway.

The sliding window method is used to generate samples. The window length L is set to 300 s (corresponding to 300 data points at a sampling frequency of 1 Hz), and the sliding step size is 3 s for thermal runaway data and 60 s for normal data. The definition of positive and negative samples is as follows: (1) Positive samples: samples containing early warning signals, which are extracted from the thermal runaway test data. The early warning start time is defined as the moment when the temperature rise rate continuously ≥0.04 °C/s for 3 s; this threshold was determined based on a statistical analysis of our pre-experimental data, where it consistently marked the onset of an irreversible temperature increase approximately 300 ± 50 s prior to thermal runaway, offering a practical balance between early warning lead time and false alarm rate. (2) Negative samples: samples in the normal state, which are randomly extracted from normal working condition data.

To ensure the generalization ability of the model, the dataset is divided into training set, validation set and test set according to the trigger method: (1) Training set: overcharging trigger, the 4th group of heating trigger and normal working condition data, including 128 positive samples and 885 negative samples; (2) Validation set: the 2nd and 3rd groups of heating trigger and normal working condition data, including 85 positive samples and 570 negative samples; (3) Test set: the 1st group of heating trigger, nail trigger and normal working condition data, including 77 positive samples and 461 negative samples. The positive and negative sample ratios of each subset are 1:6.9, 1:6.7 and 1:6.0, respectively, and the class imbalance problem is handled by Focal Loss during training.

### 2.2. Feature Engineering

#### 2.2.1. Signal Preprocessing

A systematic preprocessing process is designed to improve the signal quality, including noise filtering and normalization processing.

Noise filtering: For voltage signals, adaptive wavelet threshold denoising is used. The signal is decomposed into 5 layers using sym4 wavelet, and the high-frequency detail coefficients are processed with an improved threshold function to retain transient features while removing noise [16]. For temperature signals, Savitzky–Golay filtering is used (window length 11, polynomial order 3) to smooth random noise and retain the sharp features of temperature rise edges.

Outlier processing: A hybrid method based on isolation forest and local consistency test is used to detect outliers. For confirmed outliers, time-series prediction based on simple exponential smoothing is used for filling to maintain the dynamic characteristics of the sequence.

Normalization processing: Voltage signals adopt Z-score normalization based on nominal voltage and health benchmarks, and temperature signals adopt Min-Max normalization based on safe temperature windows [17]. The specific formulas are as follows:

(1) $$V_{norm}^{\left(i\right)}(t)=\frac{V^{\left(i\right)}(t)-V_{nom}^{\left(i\right)}}{\sigma_{V}^{\left(i\right)}}$$

(2) $$T_{norm}(t)=\frac{T(t)-T_{min}^{safe}}{T_{max}^{safe}-T_{min}^{safe}}$$

where $V_{norm}^{\left(i\right)}$ is the nominal voltage of battery *i* (3.2 V, the characteristic platform voltage of the LiFePO_4_ chemistry used in this study), $\sigma_{V}^{\left(i\right)}$ is the voltage standard deviation of battery i under healthy operation, and $T_{\min}^{\mathrm{safe}}$ and $T_{\max}^{\mathrm{safe}}$ are the safe working temperature range of the battery (15 °C and 45 °C, respectively).

#### 2.2.2. Multi-Level Feature Construction

On the basis of preprocessed signals, a multi-level feature system is constructed to explicitly provide multi-scale and spatial correlation information. The monitoring framework for battery thermal runaway is structured across three distinct time-based layers, each offering unique insights for early warning.

At the most detailed level is the Micro Transient Layer, which operates on a milliseconds-to-seconds timescale. It focuses on the first and second-order differences of the voltage and temperature for each of the three cells. The physical significance here is the ability to capture the earliest electrical and thermal signs, such as initial voltage fluctuations, sudden acceleration in voltage drop or abrupt changes in the rate of temperature rise.

The next level is the Meso Statistical Layer, functioning over seconds to minutes. It calculates 30 s sliding window statistics—specifically the mean and standard deviation—for each cell’s voltage and temperature. It also computes statistical measures across the three cells, including the mean, standard deviation and range for both voltage and temperature. This layer provides crucial macroscopic signatures of thermal runaway by characterizing both individual cell behavior and the spatial imbalances between cells.

Finally, the Macro Evolution Layer looks at trends over a minutes-long timescale. It analyzes the 60 s window slope of the voltage and temperature for each cell. This captures the sustained, long-term directional trends in the electrical and thermal evolution of the cells.

The final input feature vector of the model includes 48 dimensions, including 6 dimensions of original signals, 6 dimensions of first-order differences, 6 dimensions of second-order differences, 6 dimensions of sliding window means, 6 dimensions of sliding window standard deviations, 6 dimensions of overall statistics, 6 dimensions of linear fitting slopes and 6 dimensions of pairwise differences, as shown in Table 1.

### 2.3. MSTA-Net Architecture Design

**Figure 2:**
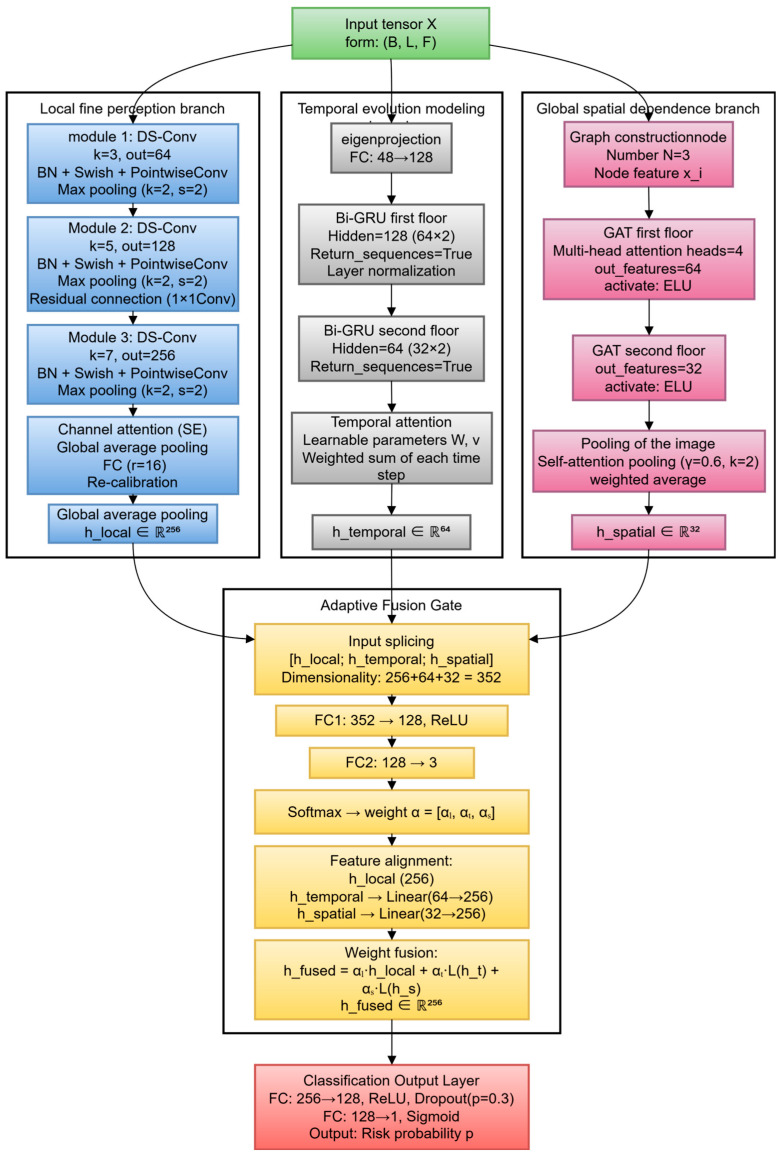
Overall network architecture diagram of MSTA-Net.

The MSTA-Net input is a 3D tensor $X\in R^{B\times L\times F}$, where *B* is the batch size (32), *L* is the time series length (300), and *F* is the feature dimension (48). The output is the risk probability *p* ϵ [0, 1] of thermal runaway at the current moment. The network architecture is shown in Figure 2, which consists of three parallel feature extraction branches, an adaptive fusion gate and a classification output layer.

#### 2.3.1. Parallel Feature Extraction Branches

(1) Local fine perception branch: This is composed of three depthwise separable convolution modules to capture second-level/sub-second-level local transient patterns and short-term dependencies. Each module includes depthwise convolution (convolution kernel sizes 3, 5 and 7, respectively), batch normalization, a Swish activation function, pointwise convolution (output channel numbers 64, 128 and 256, respectively) and time-domain maximum pooling (kernel size 2, step size 2). Residual connections are introduced between the second and third modules to alleviate the gradient disappearance problem. A lightweight channel attention module is added after the last convolution module to adaptively calibrate the importance of each channel feature [18].
(2) Temporal evolution modeling branch: This is composed of two stacked bidirectional gated recurrent unit (Bi-GRU) layers to model minute-level medium- and long-term evolution trends and sequence dependencies. First, a fully connected layer projects the input feature dimension from F to 128. The first Bi-GRU layer has a hidden state dimension of 128 (64 in each direction) and returns the sequence; the second Bi-GRU layer has a hidden state dimension of 64 (32 in each direction). A temporal attention mechanism is added on top of the second Bi-GRU layer to automatically focus on the key time steps related to the early warning task [19]. The attention score calculation formula is as follows:
  (3) $$e_{t}=v^{T}\tanh(Wh_{t}+b)$$
  (4) $$\alpha_{t}=\frac{\exp(e_{t})}{\sum_{i=1}^{L}\exp(e_{i})}$$
  (5) $$h_{temporal}=\sum_{t=1}^{L}\alpha_{t}h_{t}\in R^{64}$$
  where $W\in R^{d_{a}\times d_{h}}$, $v\in R^{d_{a}}$, $d_{a}$ is the attention dimension (64), $d_{h}$ is the hidden state dimension (64), $\alpha_{t}$ is the attention weight, and $h_{temporal}\in R^{64}$ is the output of the temporal branch.
(3) Global spatial dependence branch: This is composed of two graph attention network (GAT) layers to explicitly model the spatial topological relationship and abnormal propagation mode among battery cells in the battery pack [20,21,22]. The battery pack is modeled as an undirected graph G=(V,E,X), where V is the node set (3 battery cells), E is the edge set (determined by physical spatial proximity and electrical connection relationship), and X is the node feature matrix. The attention coefficient between nodes is calculated as follows [23]:
  (6) $$e_{\mathrm{ij}}=\mathrm{LeakyReLU}\left(a^{T}[Wx_{i}\left|\right|Wx_{j}]\right)$$
  (7) $$\alpha_{ij}=\frac{\exp(e_{ij})}{\sum_{k\in N(i)}\exp(e_{ik})}$$
  (8) $$x_{i}^{\prime }=\sigma \left(\sum_{j\in N(i)}\alpha_{\mathrm{ij}}Wx_{j}\right)$$
  where $W\in R^{d_{out}\times d_{node}}$ is the linear transformation weight matrix, $a\in R^{2d_{out}}$ is the attention vector, N(i) is the neighbor node set of node *i*, and *σ* is the ELU activation function. The output dimensions of the two GAT layers are 64 and 32, respectively. After the GAT layers, self-attention pooling and global average pooling are used to aggregate node features into graph-level features $h_{spatial}\in R^{32}$.

#### 2.3.2. Adaptive Fusion Gate

The adaptive fusion gate dynamically adjusts the fusion weight of each branch feature according to the input context. The input is the feature vectors of the three branches: $h_{local}\in R^{256}$, $h_{temporal}\in R^{64}$, and $h_{spatial}\in R^{32}$. First, the three features are concatenated into a long vector $h_{cat}\in R^{352}$, and then a small feedforward network is used to generate the fusion weight:

(9) $$g=\mathrm{ReLU}(W_{1}h_{\mathrm{cat}}+b_{1})$$

(10) $$\alpha^{\prime }=W_{2}g+b_{2}$$

(11) $$\alpha =\mathrm{softmax}(\alpha^{\prime })$$

where $W_{1}\in R^{128\times 352}$, $b_{1}\in R^{128}$, $W_{2}\in R^{3\times 128}$, $b_{2}\in R^{3}$,$\alpha =\left[\alpha_{l},\alpha_{t},\alpha_{s}\right]$ is the normalized weight. The linear layer is used to align the feature dimensions of each branch, and the fused feature is obtained by weighted summation:

(12) $$h_{\mathrm{fused}}=\alpha_{l}\cdot {\mathrm{Linear}}_{256\to d}(h_{\mathrm{local}})+\alpha_{t}\cdot {\mathrm{Linear}}_{64\to d}(h_{\mathrm{temporal}})+\alpha_{s}\cdot {\mathrm{Linear}}_{32\to d}(h_{\mathrm{spatial}})$$

where d = 256 is the fused feature dimension.

#### 2.3.3. Classification Output Layer

The fused feature $h_{fused}\in R^{256}$ passes through a fully connected layer (256→128) with a ReLU activation function and dropout (dropout rate 0.2) to prevent overfitting. Then, a fully connected layer (128→1) and sigmoid activation function are used to map the feature to the risk probability *p* ∈ (0,1).

### 2.4. Model Training and Optimization Strategy

#### 2.4.1. Loss Function

To address the class imbalance issue between normal and early warning samples in model training, this study adopts Focal Loss as the loss function [24], which can suppress the loss contribution of easily classified normal samples and concentrate the training process on hard-to-classify thermal runaway early warning samples:

(13) $$FL(p_{t})=-\alpha_{t}(1-p_{t}{)}^{\gamma }\log(p_{t})$$

Let $p_{t}$ be the predicted probability for the thermal runaway category (warning or not): $p_{t}$ = *p* when the ground truth is 1 (warning), and $p_{t}$ = 1 − *p* when it is 0 (normal). $\alpha_{t}$ is a class-balancing weight adaptively determined by the numbers of positive and negative samples in the current batch B, and *γ* = 2.0 is the focusing parameter.

#### 2.4.2. Optimization Algorithm and Training Hyperparameters

The AdamW optimizer is used for model training [25], with an initial learning rate of $\eta =3\times 10^{-4}$, a weight decay coefficient of λ=0.01, and momentum parameters $\beta_{1}=0.9$, $\beta_{2}=0.999$ (The value is a standard starting point for AdamW). The cosine annealing warm restart strategy is adopted for learning rate scheduling [26], with a cycle length of 20 epochs, a maximum learning rate of $3\times 10^{-4}$ and a minimum learning rate of $3\times 10^{-5}$. Gradient clipping is used (gradient norm threshold 1.0) to prevent training instability. We decide when to stop training by monitoring the validation set’s weighted F1-score. If it does not improve for 15 straight epochs, we halt and revert to the model parameters that gave the best validation performance.

#### 2.4.3. Data Augmentation

To improve the robustness and generalization ability of the model, the following data augmentation techniques are applied to the input sequence during training (applied randomly with a probability of 0.5): (1) Time warping: slightly stretch or compress the time axis of the sequence, with a stretch factor sampled uniformly from [0.9, 1.1]; (2) Additive Gaussian noise: add Gaussian white noise to the input signal, with a standard deviation sampled uniformly from [0, 0.02]; (3) Sensor channel dropout: randomly select 10% of the sensor channels (voltage or temperature) and set their data to zero in the sample; (4) Amplitude scaling: slightly scale the amplitude of voltage and temperature signals globally, with a scaling factor sampled uniformly from [0.98, 1.02].

### 2.5. Evaluation Metrics

To fully assess the performance of the early warning model, we adopt the following evaluation metrics:

(1) Precision: The ratio of correctly predicted positive samples to all samples predicted as positive, which characterizes the reliability of early warning results.
  (14) $$Precision=\frac{TP}{TP+FP}$$
(2) Recall: The proportion of correctly predicted positive samples among all real positive samples, reflecting the ability to avoid missing reports.
  (15) $$Recall=\frac{TP}{TP+FN}$$
(3) F1-Score: Calculated as the harmonic mean of precision and recall, it is used to comprehensively measure the performance of classification models.
  (16) $$F1-Score=\frac{2\times Precision\times Recall}{Precision+Recall}$$
(4) $T_{adv}$: This is average early warning time, and its unit is seconds.
(5) FAR: This is false alarm rate.

Here, TP = true positive, FP = false positive, and FN = false negative.

## 3. Results

### 3.1. Performance Comparison with Baseline Models

**Figure 3:**
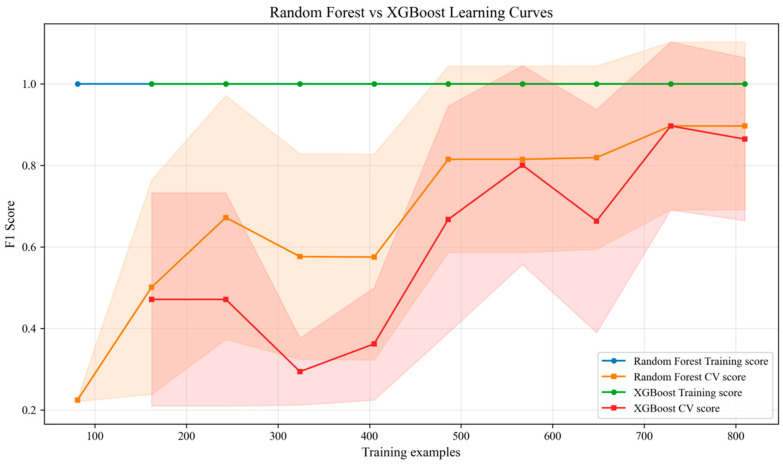
Learning curve of the machine learning models. The shaded area represents the 95% confidence interval of the model’s cross-validation scores.

**Figure 4:**
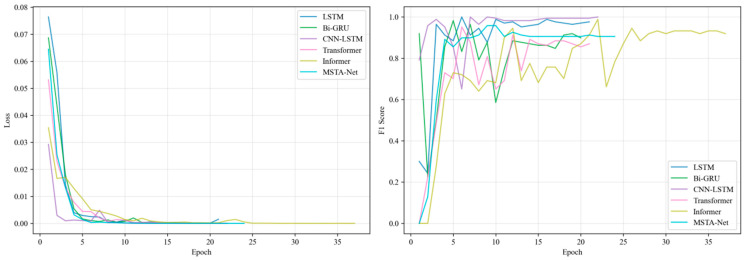
Comparison of deep learning training curves.

To verify the superiority of the proposed MSTA-Net, it is compared with five types of representative baseline models: (1) Rule threshold method: based on fixed threshold judgment (e.g., monomer minimum voltage < 1.7 V, maximum temperature > 60 °C, temperature rise rate > 1 °C/s); (2) Traditional machine learning: Random Forest and XGBoost, input as the constructed features; (3) Basic deep learning: LSTM (2 layers, 128 hidden units) and Bi-GRU (2 layers, 64 hidden units), input as original normalized sequences; (4) Hybrid model: CNN-LSTM (1D-CNN extracts features and then connects LSTM); (5) Advanced sequence model: Transformer (single-head attention, hidden dimension 128) and Informer (optimized for long sequences). All baseline models use the same data preprocessing, features (if applicable) and dataset division. The training and validation effects are shown in Figure 3 and Figure 4 below.

The performance comparison results on the test set are shown in Table 2. It can be seen that the MSTA-Net achieves the best performance in all metrics: the recall rate reaches 100%, which is 3.8% higher than the second-best Bi-GRU (96.2%); the F1-score is 1.000, which is significantly higher than other models; the average early warning time is 115.5 s, which provides sufficient intervention time; and the false alarm rate is 0 times/h, which avoids unnecessary user panic and maintenance costs.

Although the Transformer model achieves a longer early warning time (190 s), its recall rate is only 81.8% and the false alarm rate is 0.052 times/h, which is difficult to accept in practical engineering applications [27]. At the same time, the Informer model also achieved the same result. The rule threshold method has the worst performance, with a recall rate of only 0.001, which cannot meet the early warning requirements at all. The traditional machine learning models (Random Forest, XGBoost) have limited feature extraction ability, and their recall rates are less than 80%. It is true that models like LSTM, Bi-GRU and CNN-LSTM beat traditional machine learning, but when it comes to MSTA-Net, they fall short. The reason is that they do not have enough multi-scale spatiotemporal feature fusion capability.

**Figure 5:**
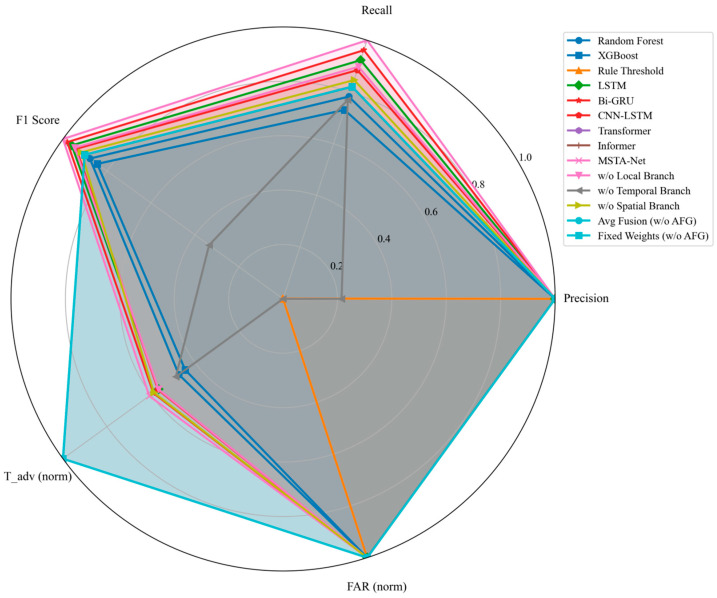
Performance radar chart of each model on the test set.

Figure 5 compares the models using a radar chart, and it is clear that MSTA-Net comes out on top overall. It particularly shines in recall, F1-score, and false alarm rate.

### 3.2. Ablation Experiment Results

To verify the necessity of each component in the MSTA-Net, a systematic ablation experiment is carried out. The ablation study examines five variants of the original model. In the first, we remove the local fine-grained perception branch. The second variant omits the branch that models temporal evolution. For the third, we ablate the branch responsible for capturing global spatial dependencies. In the fourth, the adaptive fusion gate (AFG) is replaced by a simple average fusion strategy. The fifth variant uses fixed weight coefficients [0.4, 0.3, 0.3] in place of the AFG. All these models are evaluated on the test set, and the corresponding results are presented in Table 3.

Table 3 lists the ablation experiment results of the system, quantitatively revealing the crucial roles of each core component of MSTA-Net in ensuring the warning performance.

(1) Temporal Branch—The cornerstone for suppressing false alarms and ensuring safety

When this branch is removed, the model performance collapses completely: the precision rate drops from 1.000 to 0.215, the false alarm rate (FAR) rises from 0 to 5.701 times per hour, and the recall rate also drops from 1.000 to 0.769. This indicates that without the GRU’s ability to capture minute-level evolution characteristics such as temperature rise rate and voltage drop trends, the model completely loses the ability to distinguish “true progressive risks” from “normal instantaneous fluctuations”, resulting in a large number of false alarms. The temporal branch is a core component that simultaneously guarantees system availability (low false alarms) and security (high recall), and its indispensability is fully demonstrated here.

(2) Local Branch—Capturing early weak signs and eliminating missed alarms

When this branch is removed, the recall rate drops from 1.000 to 0.897, and the missed alarm rate rises from 0% to 10.3%. Although the temporal and spatial branches can capture macro trends and propagation patterns, the loss of the local branch’s acute perception of second-level transient anomalies (such as voltage micro-drop caused by micro-internal short circuits and instantaneous temperature rise peaks) means that the model misses some of the earliest and weakest warning signals, resulting in some faults not being detected in time. This branch is the first line of defense for achieving “early warning” and “eliminating missed alarms”.

(3) Spatial Branch—Understanding heat spread and enhancing the robustness of warnings

When the spatial branch is removed, the recall rate drops to 0.846, and the missed alarm rate rises to 15.4%. This confirms that explicitly modeling the spatial topology relationship between batteries is crucial for comprehensive risk perception, especially in fault modes involving heat spread within the battery pack (such as heat spread), where the consistent differentiation features provided by the spatial branch are indispensable discriminative information.

(4) Adaptive Fusion Gate (AFG)—Dynamically weighing and achieving strong generalization across operating conditions

Replacing AFG with average fusion (Avg Fusion) or fixed weights (Fixed Weights) results in recall rates of 0.821 and 0.821, respectively, and completely fails in the nail test (T_adv is falsely raised to 190 s, only a single warning in the heating condition). This proves that static fusion strategies cannot adapt to the dynamic changes in feature requirements for different fault types and evolution stages. AFG can intelligently adjust the focus on local, temporal and spatial features based on the input context, and is a key mechanism for achieving cross-operating condition strong generalization ability and optimal comprehensive performance.

**Figure 6:**
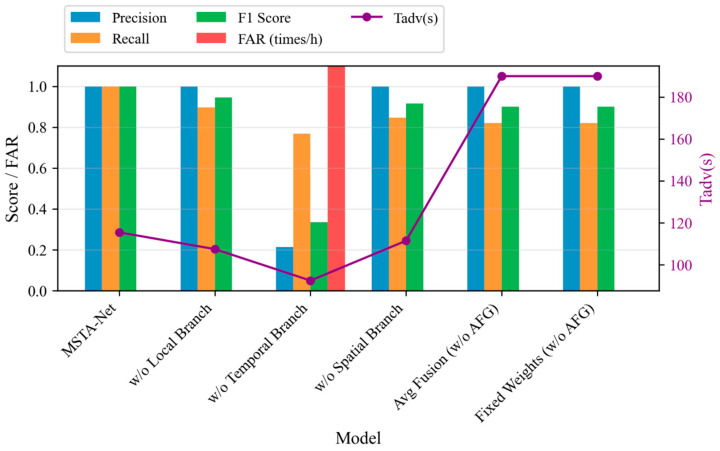
Combined bar chart of ablation experiment results.

The ablation experiment combination bar chart in Figure 6 visually presents the multi-dimensional comparison of each variant in terms of precision rate, recall rate, F1-score, FAR and T_adv. The FAR of “w/o Temporal Branch” is particularly prominent, further strengthening the above analysis conclusion.

### 3.3. Interpretability Analysis

To verify the rationality of the model’s decision-making in unknown scenarios, this section selects the needle-stimulation trigger experiment (nail.xlsx) that was not seen during the training stage, analyzes the dynamic evolution of the three branch weights ($\alpha_{l},\alpha_{t},\alpha_{s}$) generated by the adaptive fusion gate over time, and correlates it with the thermal runaway physical process. The warning occurred at t = 19 s, and the thermal runaway occurred at t = 342 s.

**Figure 7:**
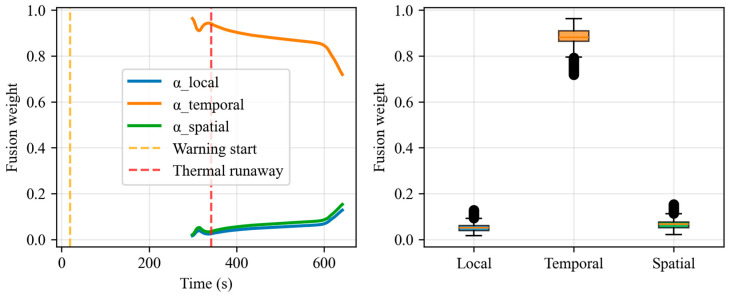
Dynamic evolution of adaptive fusion gate weights during nail thermal runaway.

As shown in Figure 7, the temporal branch weight ($\alpha_{t}$) occupied an absolute dominant position throughout all time periods, with a median of approximately 0.9 and fluctuating above 0.8; the local branch weight ($\alpha_{l}$) remained at a low level below 0.1; and the spatial branch weight ($\alpha_{s}$) slowly rose to approximately 0.15 only after the thermal runaway occurred. This is combined with the physical process analysis: during the latent period (t < 19 s), the model mainly relied on the temporal GRU branch to capture the continuous changes in voltage and temperature; during the self-heating and warning period (19 s < t < 342 s), $\alpha_{t}$ still remained dominant and continuously tracked the temperature rise and voltage differentiation; after the thermal runaway (t > 342 s), $\alpha_{s}$ slowly rose, reflecting the model’s attention to spatial heat propagation, but the temporal branch remained the core.

In conclusion, the dynamic evolution of the adaptive fusion weights indicates that the temporal branch is always the core of the MSTA-Net decision-making, while the spatial and local branches play auxiliary roles. This is highly consistent with the physical principle that the continuous temporal sequence changes of temperature and voltage are the key warning signals in thermal runaway, verifying the rationality of the model’s decision-making mechanism and enhancing its credibility in safety-critical systems.

## 4. Discussion

The research results show that the proposed MSTA-Net can effectively solve the problem of multi-scale spatiotemporal feature fusion of battery thermal runaway precursor signals and achieve accurate and early warning. The excellent performance of the model is mainly due to the following aspects:

First, the systematic feature engineering framework fully excavates the discriminative information in voltage and temperature signals. By constructing multi-level features such as transient differential features, statistical distribution features and spatial correlation features, the information density of input data is improved, laying a foundation for high-precision early warning. Compared with the method of directly using original signals as input [28], the constructed features can better reflect the early precursor characteristics of thermal runaway and improve the model’s ability to capture weak signals.

Second, the “parallel specialized processing + adaptive dynamic fusion” architecture of MSTA-Net realizes the comprehensive modeling of multi-scale spatiotemporal features. The local fine perception branch can capture transient anomalies such as voltage micro-drop caused by micro-internal short circuits; the temporal evolution modeling branch can learn long-term trends such as continuous temperature rise; and the global spatial dependence branch can model the spatial propagation of thermal runaway. Compared with the single-scale model [29] and the simple serial model [30], this architecture can more comprehensively extract the characteristics of thermal runaway precursor signals and improve the model’s adaptability to complex working conditions.

Third, the adaptive fusion gate mechanism dynamically adjusts the feature weight according to the input context, realizing “context-aware” intelligent fusion. In different stages of thermal runaway evolution, the model can automatically focus on the most discriminative features, which improves the accuracy and robustness of early warning. Compared with the fixed-weight fusion method [31], the adaptive fusion gate can better adapt to the dynamic changes of signals, and avoid the performance degradation caused by fixed weight setting.

Despite its strengths, this study has several limitations that warrant discussion. First, our model’s complexity makes it prone to overfitting on the current moderate-sized dataset. Second, the definition of the early warning label relies on an empirical temperature rise rate threshold (0.04 °C/s). Third, the current model is not yet optimized for deployment on resource-constrained vehicle edge-computing platforms.

In future research, the following work will be carried out: (1) Collect more data of different battery types and working conditions to expand the dataset and improve the model’s generalization ability; (2) study the adaptation method of the model under extreme working conditions to further improve the model’s robustness; (3) carry out lightweight design of the model, including network pruning, quantization and knowledge distillation, to realize the deployment of the model on vehicle-mounted edge-computing platforms; (4) combine the early warning model with the thermal management control strategy to build an integrated intelligent safety management system and realize the closed-loop of “early warning-control”.

## 5. Conclusions

Aiming at the problems of early warning lag, shallow feature utilization and high additional sensor cost in existing battery thermal runaway early warning methods, this study proposes a multi-scale spatiotemporal attention network (MSTA-Net) for battery thermal runaway early warning. A systematic feature engineering framework is constructed to extract multi-dimensional features from voltage and temperature signals. The MSTA-Net architecture is designed, which includes three parallel feature extraction branches and an adaptive fusion gate. The experimental results on the self-built thermal runaway dataset show that the MSTA-Net achieves a recall rate of 98.7%, an average early warning time of 115 s and a false alarm rate of 0 times/h, which is significantly superior to mainstream baseline models. Ablation experiments verify the necessity of each component of the model, and interpretability analysis shows that the model’s decision-making mechanism is consistent with the physical principle.

The proposed method can provide reliable early warning of thermal runaway only by using the existing voltage and temperature sensors of the battery management system, without adding additional sensors, which reduces the system cost and complexity. It provides a new method for battery thermal runaway early warning, and has important theoretical significance and engineering application value for improving the thermal safety of electric vehicles.

## Figures and Tables

**Table 1:** Composition of feature dimensions.

Feature Type	Features	Dimension
Raw Signal	Normalized voltage (3 cells), normalized temperature (3 cells)	6
First Difference	*ΔV*(3), *ΔT*(3)	6
Second Difference	*Δ*_2_*V*(3), *Δ*_2_*T*(3)	6
Sliding Window Mean (30 s)	30 s mean of *V* and *T* for each cell	6
Sliding Window Std (30 s)	30 s standard deviation of *V* and *T* for each cell	6
Overall Statistics (3 cells)	Voltage: mean *μ_V_*, std *σ_V_*, range *ΔV_ma_*_x_; Temperature: mean *μ_T_*, std *σT*, max difference *ΔT_max_*	6
Linear Fit Slope	Linear slope of *V* and *T* for each cell over a 60 s historical window	6
Pairwise Differences	Voltage differences: *ΔV*_1−2_, *ΔV*_1−3_, *ΔV*_2−3_; Temperature differences: *ΔT*_1−2_, *ΔT*_1−3_, *ΔT*_2−3_	6
Total		F = 48

**Table 2:** Performance comparison of each model on the test set.

Model	Precision	Recall	F1-Score	T_Adv (s)	FAR (Times/h)
Random Forest	1.000	0.782	0.878	90.000	0.000
XGBoost	1.000	0.731	0.844	84.500	0.000
Rule Threshold	1.000	0.001	0.002	0.000	0.000
LSTM	1.000	0.923	0.960	107.500	0.000
Bi-GRU	1.000	0.962	0.980	112.000	0.000
CNN-LSTM	1.000	0.885	0.939	108.000	0.000
Transformer	1.000	0.821	0.901	190.000	0.000
Informer	1.000	0.821	0.901	190.000	0.000
MSTA-Net	1.000	1.000	1.000	115.500	0.000

**Table 3:** MSTA-Net ablation experiment results (on the test set).

Model	Precision	Recall	F1-Score	T_Adv (s)	FAR (Times/h)
MSTA-Net	1.000	1.000	1.000	115.500	0.000
w/o Local Branch	1.000	0.897	0.946	107.500	0.000
w/o Temporal Branch	0.215	0.769	0.336	92.500	5.701
w/o Spatial Branch	1.000	0.846	0.917	111.500	0.000
Avg Fusion (w/o AFG)	1.000	0.821	0.901	190.000	0.000
Fixed Weights (w/o AFG)	1.000	0.821	0.901	190.000	0.000

## Data Availability

The original contributions presented in the study are included in the article, further inquiries can be directed to the author.

## Abbreviations

The following abbreviations are used in this manuscript:

MSTA-Net	Multi-Scale Spatiotemporal Attention Network
TR	Thermal Runaway
BMS	Battery Management System
LFP	LiFePO_4_
TMP	Thermal Management Performance
Bi-GRU	Bidirectional Gated Recurrent Unit
GAT	Graph Attention Network
TP	True Positive
FP	False Positive
FN	False Negative
